# Genome-Wide Analysis of Alternative Splicing during Dendritic Cell Response to a Bacterial Challenge

**DOI:** 10.1371/journal.pone.0061975

**Published:** 2013-04-17

**Authors:** Raquel Rodrigues, Ana Rita Grosso, Luís Moita

**Affiliations:** Instituto de Medicina Molecular, Faculdade de Medicina, Universidade de Lisboa, Lisboa, Portugal; INSERM, France

## Abstract

The immune system relies on the plasticity of its components to produce appropriate responses to frequent environmental challenges. Dendritic cells (DCs) are critical initiators of innate immunity and orchestrate the later and more specific adaptive immunity. The generation of diversity in transcriptional programs is central for effective immune responses. Alternative splicing is widely considered a key generator of transcriptional and proteomic complexity, but its role has been rarely addressed systematically in immune cells. Here we used splicing-sensitive arrays to assess genome-wide gene- and exon-level expression profiles in human DCs in response to a bacterial challenge. We find widespread alternative splicing events and splicing factor transcriptional signatures induced by an *E. coli* challenge to human DCs. Alternative splicing acts in concert with transcriptional modulation, but these two mechanisms of gene regulation affect primarily distinct functional gene groups. Alternative splicing is likely to have an important role in DC immunobiology because it affects genes known to be involved in DC development, endocytosis, antigen presentation and cell cycle arrest.

## Introduction

The immune system protects the host from continuous environmental challenges. In vertebrates, it relies on innate and adaptive components to achieve efficient and specific responses [1]. Dendritic cells (DCs) are involved in the initiation of innate and adaptive immunity and play a key role controlling the magnitude and quality of adaptive immune responses [2].

Immature DCs decode and integrate signals by directly recognizing microbial components or by receiving signals formulated by other players of the innate immune system that is exposed to microbes, and convey this information to adaptive immune cells [3]. To elicit such a response DCs go through a complex maturation process from antigen-capturing into antigen-presenting cells (APCs), undergoing changes that include remodelling in cytoskeleton and acquisition of high mobility [4], loss of endocytic/phagocytic receptors, secretion of chemokines [5], [6], [7], up-regulation of costimulatory molecules [8], translocation of MHCII compartments to cell surface [9] and secretion of cytokines that differentiate and polarize the immune effectors that have been attracted by the chemokines [10]. DC maturation is also a terminal differentiation process marked by shutdown of the cell cycle followed by programmed cell death [11], [12], [13], [14].

The immune system relies on the generation of highly diverse detection, transduction and effector mechanisms and in the ability of individual cells to rapidly adapt and respond to changing environmental conditions [15]. Such diversity and flexibility of function require several mechanisms of gene regulation. Much attention and effort has focused on understanding the role of transcriptional regulation in the immune system in recent years, but it is clear that other mechanisms of gene regulation must operate for effective immune responses to take place. One such mechanism with a widespread role in gene regulation is alternative pre-mRNA splicing (AS), which permits the production of multiple functionally distinct proteins from a single gene [16].

The conclusion of the Human Genome Project revealed the presence of much fewer coding genes than formerly estimated [17], supporting the hypothesis of a major role for AS as a generator of complexity. Recent studies estimate that around 95% of human primary transcripts can undergo AS [18], [19] and that the numbers are particularly elevated in the immune and nervous systems, in agreement with their complexity and need for plasticity [16], [20]. Various examples of AS in the immune system have been observed where different isoforms of the same gene are responsible for different functional outcomes. CD45 and cytotoxic T-lymphocyte antigen 4 (CTLA4) have been particularly well documented. In these cases, alternative splicing has been shown to generate either soluble forms of the molecules or molecules with altered protein–protein interactions significantly changing the threshold of T-cell activation [16]. Furthermore several immune-related diseases and metastatic spread of tumors have been connected to alterations to the normal splicing events. This observation in itself provides evidence of the importance of AS in modulating the function of the immune system ([16] and references herein). Nevertheless there have been only a few systematic studies to determine which genes are regulated at the alternative splicing level in pathologies [19], [21], [22], [23] or in response to an immunological challenge [24].

Reflecting the scarce number of systematic studies of alternative splicing in the context of an immune response, there are no previous reports focusing on dendritic cells in response to an immune challenge. To systematically explore the role of alternative splicing in the immunobiology of DCs, we used the splicing-sensitive GeneChip® Human Exon 1.0 ST Arrays to assess gene- and exon-level expression profiling at the whole genome scale. We found that distinct yet sometimes overlapping functional groups were observed in differentially expressed genes (DEG) and alternative splicing events (ASE), suggesting that AS is a broad mechanism that operates in concert with regulated transcription. We identified a splicing factor specific signature with tight regulation in these cells. Using qPCR/RT-PCR we have validated all tested transcriptional events and 60% of the alternative splicing events, which is in good accordance with previous studies using the same array platform. Genes identified to undergo AS may be involved in important functions in DCs, such as regulation of DC development, endocytosis, antigen presentation or cell cycle arrest. Functional analysis suggests that the immune response genes are mostly regulated by at the transcriptional level, whereas AS seems to tune general cellular functions.

## Materials and Methods

### Ethics Statement

All subjects whose samples were used for the validation of the array results gave written informed consent for blood sampling and processing. The study was approved by the Ethical Board of the Faculty of Medicine of the University of Lisbon. The samples hybridized to the microarrays have been previously published [26].

### Cell Culture

DCs for hybridization in the microarrays were obtained as follows: Elutriated human monocytes (Advanced Biotechnology Inc., Columbia, MD) were grown in RPMI with 10% fetal bovine serum (Life Technologies, Paisley, UK) supplemented with 1000 U/ml GM-CSF (R&D Systems, Minneapolis, MN) and 1000 U/ml IL-4 (R&D Systems Minneapolis, MN) for 7 days in 24 well plates (10^6^ cells in 1 ml medium/well), and fed every 2 days after plating [25]. On day 7, DCs were harvested and aliquoted into 100 mm plates at 10^7^ cells/plate/timepoint and incubated at 37°C for 60 min. *E.coli* SD54 (ATCC, Middlesex, UK) [multiplicity of infection (MOI) 5∶1] was then added to the DCs and cultured for 4 hours (timepoint 1 – T1), 8 hours (timepoint 2 – T2) and 18 hours (timepoint 3 – T3). Control DCs (non-stimulated cells) were collected at equivalent timepoints. Stimulated cells showed mature DC dendrite formation, were less adherent at 24 hours, expressed typical DC markers (CD83, CD86), and stimulated allogeneic T cell proliferation at DC:T ratios of 1∶1000–1∶10 [26].

DCs for quantitative real-time polymerase chain reaction (qPCR) and reverse-transcription polymerase chain reaction (RT-PCR): Peripheral blood mononuclear cells were isolated from buffy coats of healthy donors by density gradient using Ficoll-Paque (GE Healthcare, Waukesha, WI). CD14^+^ monocytes were isolated, magnetically labeling them with CD14 microbeads (Miltenyi Biotec, Bergisch Gladbach, Germany) followed by cell sorting in MACS separation columns (Miltenyi Biotec, Bergisch Gladbach, Germany), according to manufacturers' protocols. These monocytes were then plated for 7 days with 10^3^ units/ml of hGM-CSF (R&D Systems, Minneapolis, MN) and IL-4 (R&D Systems, Minneapolis, MN), thus obtaining monocyte derived dendritic cells. On day 7, DCs were harvested and aliquoted in 24 well plates at 10^6^ cells/well/timepoint and incubated with *E.coli* fixed in 4% PFA (MOI 20∶1) for 4 hours (T1), 8 hours (T2) and 18 hours (T3). Non-stimulated cells were also harvested as controls (T0). Total RNA was extracted with Trizol® (Molecular Research Center Inc., Cincinnati, OH), using standard procedures. Approximately 1 µg of total RNA from stimulated and control DCs from a pool of donors was reverse transcribed with SuperScript™ II (Life Technologies, Paisley, UK), using Oligo(dT)12–18 (Life Technologies, Paisley, UK) to prime reverse transcription.

### GeneChip® Human Exon 1.0 ST Arrays

The data discussed in this publication have been deposited in NCBI's Gene Expression Omnibus [27] and are accessible through GEO Series accession number GSE42561 (http://www.ncbi.nlm.nih.gov/geo/query/acc.cgi?acc=GSE42561).

At each timepoint, control and stimulated DCs were harvested and lysed using TRIzol® (Molecular Research Center Inc., Cincinnati, OH). Total RNA was isolated, labeled and prepared for hybridization to the commercial GeneChip® Human Exon 1.0 ST Array (Affymetrix, Inc., Santa Clara, CA) using standard methods [28]. Hybridization was carried out according to Affymetrix standard procedures, and arrays were scanned on Affymetrix scanners. Microarray data pre-processing and summarization was performed using the AltAnalyze application [29], which includes several statistical methods for rigorous data filtering, summarization and determination of differential splicing. Briefly, low quality probe sets were removed according to detection above background (DABG) p-value and absolute expression values. Next, probe sets were summarized with RMA algorithm and using the AltAnalyze annotations, derived from Ensembl [30] and UCSC databases [31]. Gene expression levels were determined using only probe sets targeting constitutive exons. Determination of overall gene level variation was assessed using linear models and empirical Bayes methods [32] as implemented in the ‘limma’ package [33]. The B-statistics gives the log odds of differential expression and it requires an ‘a priori’ value for the estimated proportion of differentially expressed genes. To determine this value, we visually inspected the volcano plot, which compares biological significance (represented by fold-changes) with statistical significance (B-values) [34], finding the value which enabled genes to be distinguished from the majority (logFC≤∼1.585) [35]. Additionally, we verified the P-values corresponding to moderated F-statistics. Using the Benjamini and Hochberg method [36], all genes selected as differentially expressed had adjusted P-values<0.05. Detection of alternative splicing variations were determined using splicing-index [37] and FIRMA approaches [38], calculating MiDAS [39] and normalized intensity p-values (p-value <0.05). We used the Molecular Signatures DataBase [40] to test enrichment of canonical pathways; BioCarta, Kegg and Reactome gene sets. Terms with p-value<0.05 for Hypergeometric distribution were selected.

#### Gene Expression Validation by qPCR

To validate Exon array results in terms of gene expression, qPCR was performed using an ABI PRISM 7500 Sequence Detection System (Applied Biosystems, Carlsbad, California). We tested 28 genes in stimulated and control DCs (Table S1). Sequence specific primers were designed, using Primer3 v0.4.0 (Whitehead Institute for Biomedical Research, Cambridge, MA) and Oligo 6.7 (Molecular Biology Insights Inc, Cascade, CO) taking in account previously described isoforms for these genes, according to ENSEMBL. A common sequence string for the isoforms was chosen, and the primers were designed to anneal there (Table S1). As endogenous control we selected the reference gene GAPDH. The conditions of the SYBR® GreenER (Life Technologies, Paisley, UK) PCR were as follows: 50°C for 2 min, 95°C for 10 min, followed by 40 cycles of 95°C for 15 s and 60°C for 1 min. The relative expression of each sample was calculated with respect to a standard calibration curve that represents a serial dilution of cDNA positive for the expression of the gene in analysis. Each sample was analyzed three times and PCR experiments were usually duplicated and included at least one non-template control well.

#### Alternative splicing validation and Criteria for selecting validation targets

Targets for AS validation were selected according to a number of conditions. The top hits were selected considering FCs and p-values. The alternative splicing events were chosen preferably when occuring in ENSEMBL exons, and whole exon gain/loss was privileged. First and last exon events were excluded, due to constrictions in primer design. Considering this criteria 12 genes were chosen for validation (Table S2). RT-PCR was applied to assess the inclusion or exclusion of exons predicted by Exon Array analysis. RT-PCR primers were designed in expressed sequences of exons flanking the target sequence (Table S2), using the primer analysis software *Oligo®* version 6.70 (Molecular Biology Insights Inc, Cascade, CO). RT-PCR was carried out with Supreme NZYTaq 2x Green Master Mix (NZYTech, Lisboa, Portugal) according to manufacturer's instructions. The reactions were run on a Unocycler thermocycler (VWR, Randor, PA) and PCR products were analyzed on GelRed (Biotium Inc., Place Hayward, CA) stained 1.5–2% agarose gels.

## Results

We used GeneChip® Human Exon Arrays from Affymetrix (Affymetrix, Inc., Santa Clara, CA) to assess ASE and DEG at the genome-wide level in DCs during an immune response to an *E.coli* challenge. These arrays measure expression levels of exons as independent objects and contain probes for all predicted exons in the human genome. The expression of most RefSeq genes is monitored by 30–40 probes distributed along the entire length of the transcript, which makes these arrays particularly effective at detecting differences in expression, when compared to other expression arrays [37]. DCs were stimulated with *E.coli* for 3 distinct periods: (T1) 4 hours, to cover early changes that occur after the bacterial challenge; (T2) 8 hours, to assess intermediate changes; and (T3) 18 hours, to measure the late response stage.

### Genome-wide analysis reveals a sustained program of gene regulation throughout DC response to *E.coli* challenge

**Figure 1:**
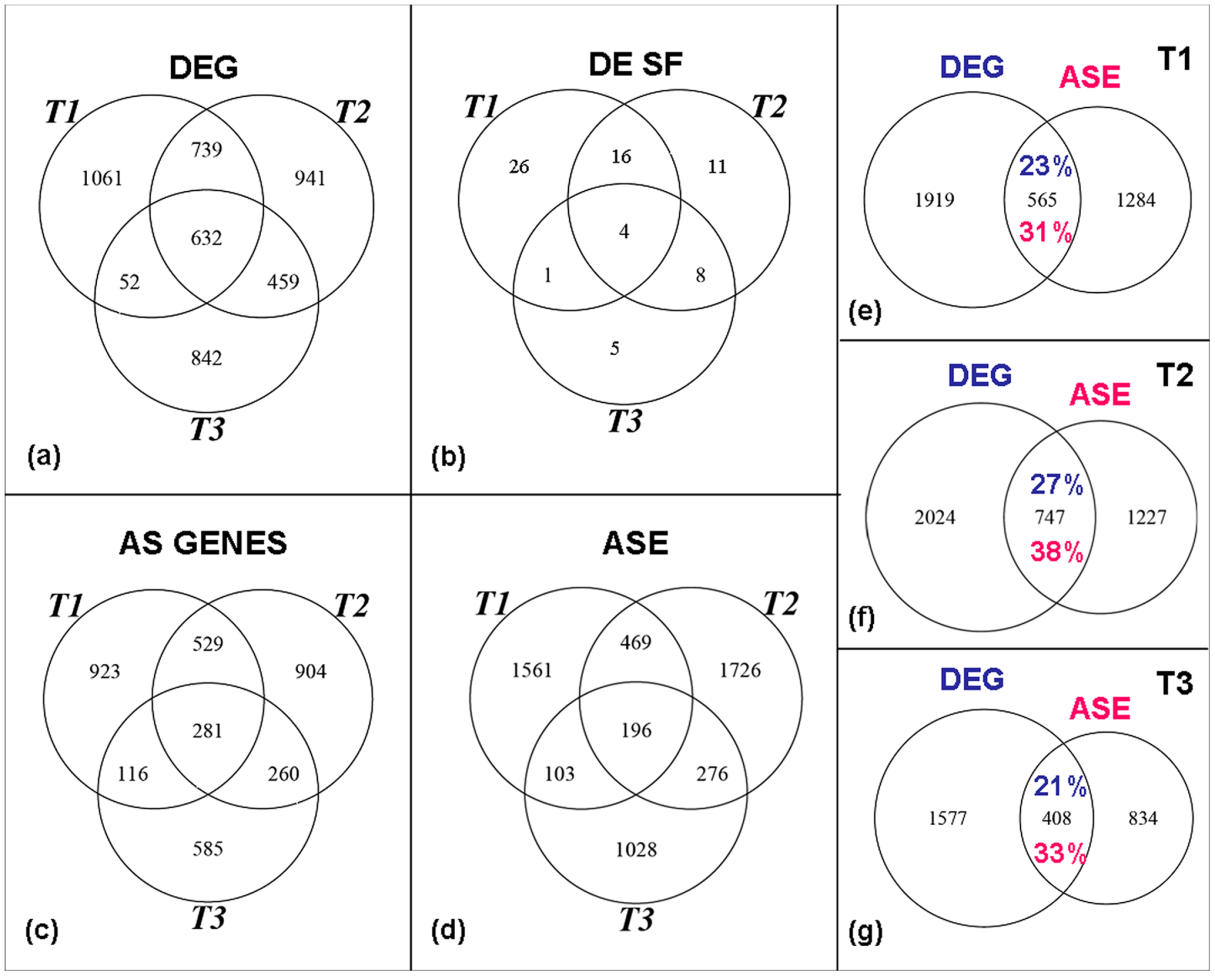
Venn diagrams of the (a) differentially expressed genes, (b) differentially expressed splicing factors, (c) alternatively spliced genes and (d) alternative splicing events in dendritic cells, according to exon array analysis, following *E.coli* challenge in the evaluated timepoints: T1 (4 h), T2 (8 h), T3 (18 h). Venn diagrams of differentially expressed genes and alternative splicing events after (e) 4 h, (f) 8 h and (g) 18 h of stimulus. (in blue: percentage of the differentially expressed genes that are also alternatively spliced; in pink: percentage of the alternatively spliced genes that are also differentially expressed).

Analysis of GeneChip® Human Exon Arrays (Affymetrix, Inc., Santa Clara, CA) was performed using strict statistical methods to assess DEG between non-stimulated and stimulated DCs at T1, T2 and T3. From the 17800 analyzed transcripts, respectively 55%, 59% and 52% were expressed in the cells after 4 h, 8 h and 18 h of bacterial challenge and correspondingly 2484, 2771 and 1985 were differentially expressed. Although the majority of highly regulated genes were upregulated at all timepoints, downregulated genes were predominant at the two earlier timepoints with 68% of the transcripts downregulated. At T3 we observed a balance with 49% of downregulation (Table 1, Table S3). The number of genes exclusively differentially expressed at each timepoint is comparable and large numbers of transcripts are consistently regulated at consecutive timepoints, as suggested by crossing the DEG at T1, T2 and T3 (Figure 1A).

**Table 1:** Differentially Expressed Genes and Alternative Splicing in DCs, upon *E.coli* challenge in the three appraised timepoints.

Timepoint	Analyzed transcripts	Expressed Transcripts	Differentially expressed genes (DEG)			AS Genes	AS Events (ASE)		
**T1 (4 h)**	**17800**	**9862 (55%)**	**2484**	**Up**	**791 (31.8%)**	**1849**	**2329**	**Exon Gain**	**820 (35%)**
				**Down**	**1693 (68.2%)**			**Exon Loss**	**1509 (65%)**
**T2 (8 h)**	**17800**	**10529 (59%)**	**2771**	**Up**	**877 (31.6%)**	**1974**	**2667**	**Exon Gain**	**870 (33%)**
				**Down**	**1894 (68.4%)**			**Exon Loss**	**1797 (67%)**
**T3 (18 h)**	**17800**	**9332 (52%)**	**1985**	**Up**	**1008 (50.8%)**	**1242**	**1603**	**Exon Gain**	**654 (41%)**
				**Down**	**977 (49.2%)**			**Exon Loss**	**949 (59%)**

AS – alternative splicing, Up – upregulated, Down – downregulated.

The samples used in the current study have been previously hybridized to HuGeneFL oligonucleotide arrays [26]. We found a 62% agreement rate between the expression trends of the reported genes with our own study (Table S4), which we consider satisfactory given the significant differences in the platforms used and in the analysis performed. Huang *et al.* [26] selected their upregulated genes according to the following criteria applied at the same time to all three donors: (i) S_i_>1.2 for ≥2 consecutive timepoints or (ii) S_i_>4 for ≥1 timepoint. Downregulated genes were selected if S_i_≤−1.4 for >4 points (details for the scoring system is available in Huang *et al.* [26] supplemental data). We determined our regulated genes not considering consecutive timepoints, but comparing non-stimulated *vs* stimulated DCs at each timepoint, and using different methods (described in Materials and Methods section).

**Figure 2:**
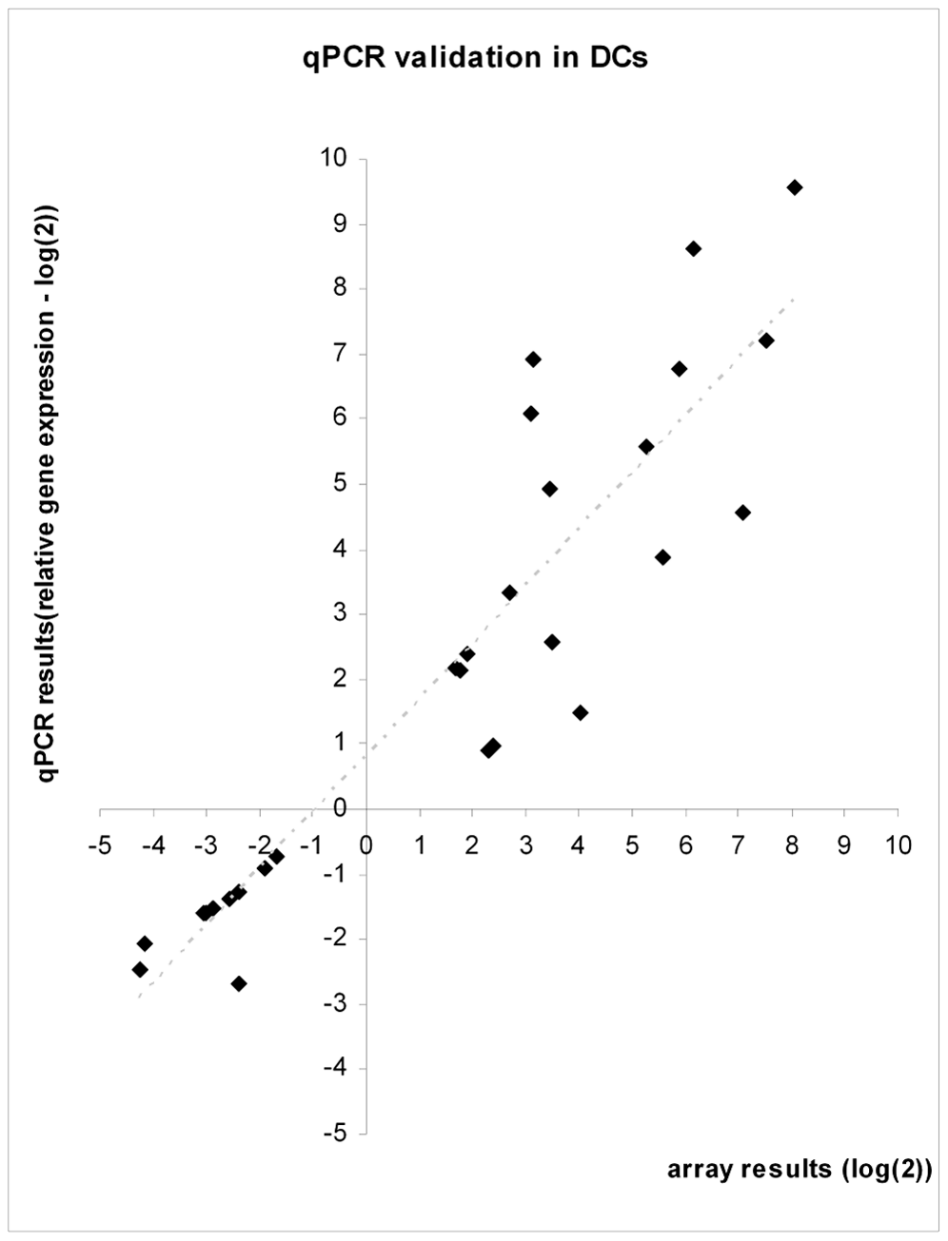
Validation of array results by quantitative real-time polymerase chain reaction. Twenty-eight differentially expressed genes, according to array analysis, were tested by qPCR in dendritic cells, comparing non-stimulated and challenged cells. Results are presented in log(2). (See Table S1 for details).

To validate our array results we produced independent DCs samples derived from monocytes of 9 donors and chose 28 genes to be tested by qPCR (Table S1). We selected 15 splicing factors out of the total 28 tested genes given our interest and subject of the current study. Also, 14 of the tested genes had previous reported expression array data [26] that matched our results in terms of tendency of regulation in response to *E.coli* challenge (Table S4). The trends for all of the results obtained in the arrays were validated by qPCR analysis (Figure 2).

### Identification of a Splicing Factor gene expression signature in DC maturation

**Figure 3:**
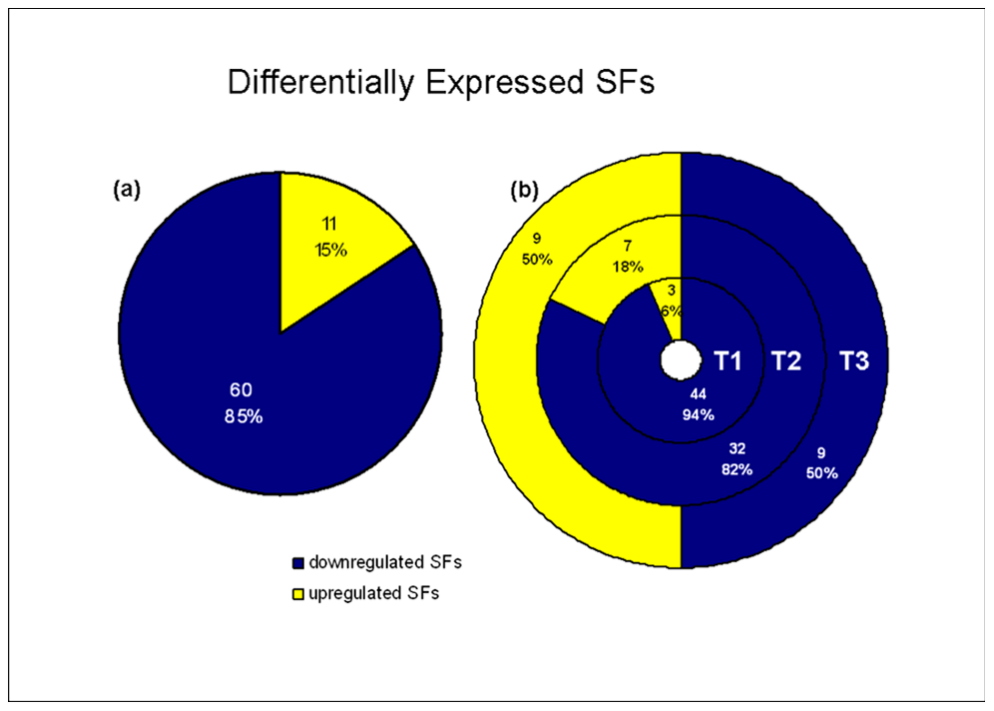
Differentially expressed splicing factors. (**a**) Overall trends of expression of the splicing factors (**b**) Trends of expression of the splicing factors across the analyzed timepoints: T1 (4 h), T2 (8 h), T3 (18 h) – percentage of the up and downregulated splicing factors.

To study the role of splicing modulators in the response of DCs to a bacterial challenge, we compiled a comprehensive list of 442 genes that have been previously connected to splicing [41] (here broadly denominated as splicing factors (SFs)) (Table S5). We then crossed this list with that of DEG in the three evaluated timepoints. Larger percentages of the SFs (62%–77%) than the generality of genes (52–59%) were expressed underlying the importance and pervasiveness of splicing regulation in DC physiology and maturation. Among the expressed SFs respectively 47, 39 and 18 were regulated at T1, T2 and T3 (Table S6). By crossing the differentially expressed SFs at the different timepoints (Figure 1B), we observe that most SFs were regulated in the early and mid stage. In whole, there were 71 differentially expressed SFs: 60 (85%) downregulated and 11 (15%) upregulated (Figure 3A, Table S6). Although most SFs were downregulated, there was a tendency for a change in trends along time: there were only 6% of the differentially expressed SFs upregulated after 4 hours of *E.coli* challenge, 18% after 8 h and 50% 18 h following the stimulus (Figure 3B). Moreover, the majority of the SFs had logFCs <2.5.

**Figure 4:**
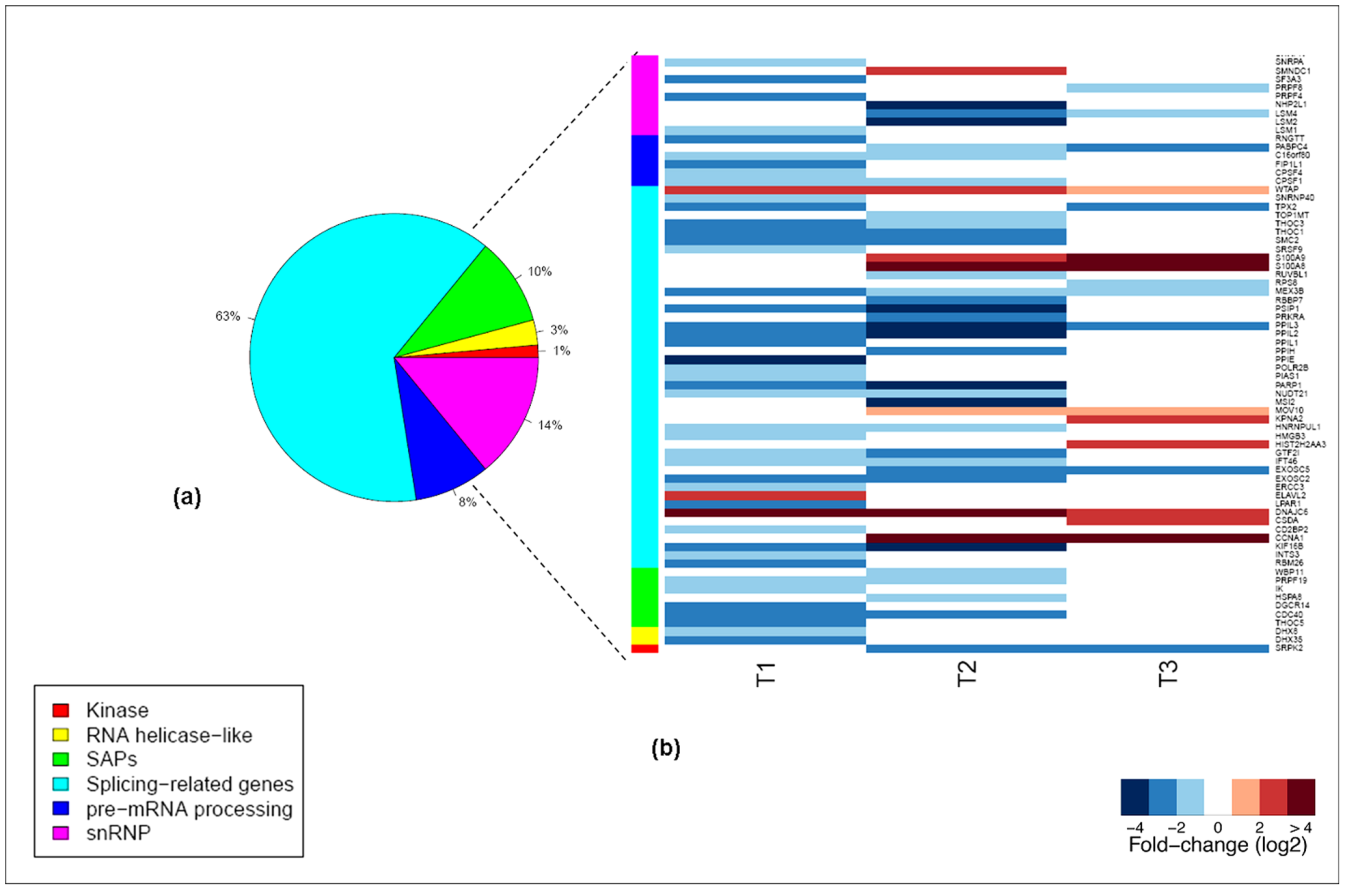
Protein families of the differentially expressed splicing factors. (**a**) Protein family distribution of the differentially expressed splicing factors; (**b**) Heatmap with the fold changes (log(2)) observed for the differentially expressed splicing factors, at the three evaluated timepoints (T1: 4 h; T2: 8 h; T3: 18 h), grouped by protein family. The genes and respective logfold changes are presented in detail in Table S6.

The differentially expressed SFs included members of distinct protein families: snRNP (14%), spliceossome associated (SAPs) (10%), pre-mRNA processing (8%), RNA helicase-like (3%), kinases (1%) and other splicing related proteins (63%) (Figure 4A). All of the SFs included in SAPs, pre-mRNA processing and RNA helicase-like families were downregulated, mostly in the early and intermediate stages of DC response to stimulus (Figure 4B).

Many of the genes selected for validation by qPCR were included in the SFs category (Table S1): 15 SFs were tested in DCs, chosen randomly to cover most SFs families, up- and downregulation, and all the ranges in FC. As previously mentioned, all were validated.

### *E.coli* challenge of DCs induces novel isoform expression

The alternatively spliced genes and splicing events between non-stimulated and stimulated DCs were assessed at T1, T2 and T3. A stringent analysis aiming to minimize false positives was performed, and the lists of genes that undergo AS comprised 13–19% of the expressed transcripts (Table 1, Table S7). At all timepoints several genes showed more than one splicing event (ASE>AS genes). The ratio of exon inclusion/exon exclusion did not change significantly during DC maturation, and there was a slight tendency to exon exclusion upon stimulus (Table 1). The Venn diagram obtained by crossing the AS genes (Figure 1C) and ASE (Figure 1D) showed, especially when compared to DEG, that the events are not as sustained across time. Furthermore we observed that 31–38% of the alternatively spliced genes are also differentially expressed. Conversely only 21–27% of the DEG are alternatively spliced (Figure 1E–G).

**Figure 5:**
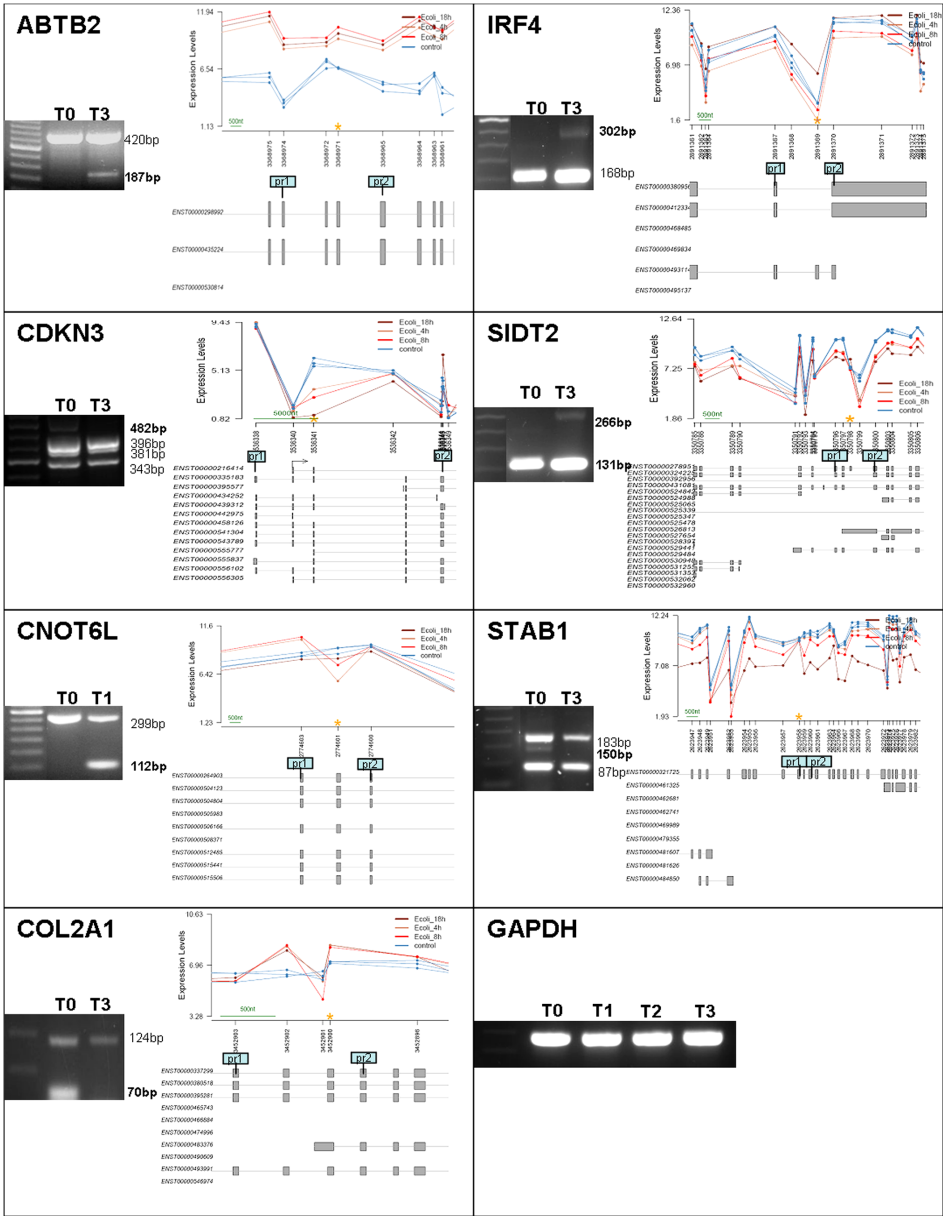
Positively validated genes with alternative splicing in dendritic cells after challenge with *E.coli*. Analysis of PCR products on GelRed stained 1.5–2% agarose gels (in some cases the figure was cropped so that the lane with the DNA ladder was adjacent to the lanes of interest) and schematic representation of the region of the gene that was tested. The yellow asterisk marks the significant probeset and the blue boxes represent the areas where the primers were designed to anneal. The same amount of cDNA was used in all RT-PCR reactions, as shown in GAPDH amplification reaction. Details are supplied in Table S2.

To validate the inclusion or exclusion of exons predicted by Exon Array analysis we tested 12 genes by RT-PCR (Table S2). To this end, primers were designed to anneal sequences expressed in both control and stimulated cells, flanking the target sequence (Table S2). We did not confirm 5 ASE out of the 12 genes chosen for validation: both of the predicted isoforms were present in stimulated and non stimulated cells in comparable amounts (ALG12, LAT2 and NSF) or only one isoform became visible in agarose gel (ARHGEF1 and PRKCI) (Figure S1). ABTB2, CDKN3, CNOT6L, COL2A1, IRF4, STAB1 and SIDT2 showed AS in DCs after *E.coli* challenge - a new isoform was evident in agarose gel (Figure 5). In summary, we obtained an estimated validation rate of 60% for the genes that undergo AS. One of the interesting findings, when analyzing RT-PCR results was the discovery of previously unreported isoforms in 8 transcripts, even in genes where we were not able to validate the AS pattern predicted by the splicing-sensitive arrays.

To investigate if alternative splicing also contributed to the regulation of splicing factors, we assessed the number of SFs that underwent signal induced AS. At all timepoints a total of 68 SFs was alternatively spliced, respectively 45 at T1, 28 at T2 and 21 at T3 (Table S8). This corresponded to 15% of the SFs expressed in the cell at T1, and 8% at T2 and T3.

In DCs, genes directly associated with the immune response are mainly regulated at the transcriptional level whereas AS fine-tunes more general cellular functions

**Figure 6:**
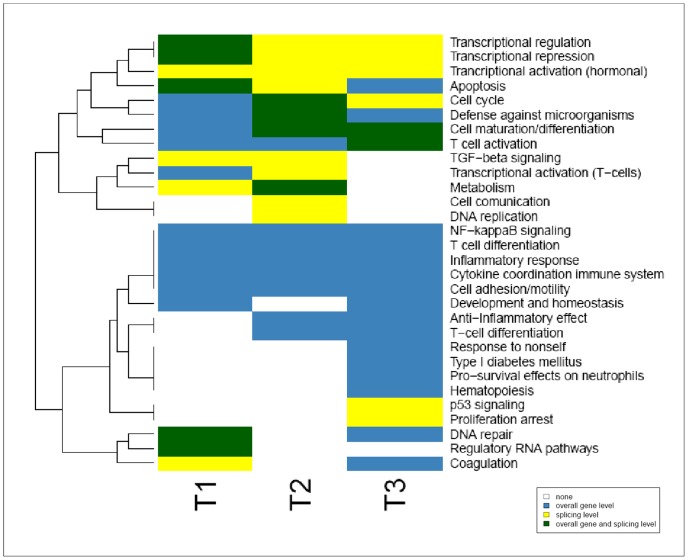
Functional Analysis of differentially expressed and alternatively spliced genes. Dynamic heatmap (T1, T2 and T3) of the 30 functional groups obtained by grouping the related terms within enriched gene sets in alternatively spliced genes (yellow), differentially expressed genes (blue) or both (green). Details are provided in Table S9.

We used the Molecular Signatures DataBase [40] to test enrichment of canonical pathways and BioCarta, Kegg and Reactome gene sets within DEG and AS genes. A total of 103 gene sets were obtained and related gene sets were then grouped, thus reaching 30 Functional Groups (Figure 6, Table S9).

### DEG

Considering the DEG, several Functional Groups were consistently regulated throughout time, in accordance with what we detected when crossing the DEG (Figure 1A): NF-kappaB signaling, T-cell differentiation, inflammatory response, cytokine coordination of the immune system and cell adhesion/motility. Most of these features directly relate to immune response Interestingly this is also the case for the two later timepoints, with Functional Groups such as anti-inflammatory effect and T-cell differentiation regulated at T2+T3 or response to non-self and hematopoiesis regulated at T3.

### ASE

Overall, the assessed alternatively spliced genes grouped in fewer exclusive Functional Groups than the DEG. The only Group that was consistently regulated at all timepoints by ASE was transcriptional activation (hormonal). This fact is in accordance to what was observed in the crossing of the genes that undergo AS (Figure 2C), where we noticed that the number of genes spliced across different timepoints is restricted. Other genes that were shaped by splicing related to TGF-beta signaling (T1+T2), cell communication and DNA replication in a mid stage, and in the latter timepoint p53 signaling and proliferation arrest.

### ASE+DEG

Transcriptional repression was assured throughout DC maturation in response to *E.coli* challenge, early on by AS and differential expression and in later timepoints by AS only. Both this mechanisms of gene regulation also acted cooperatively to control crucial functions such as apoptosis, metabolism or DNA repair. A considerable set of the Functional Groups regulated by both mechanisms were in earlier phases managed by differences in expression, and latter on AS also contributed to their shaping: cell cycle, cell maturation/differentiation, defense against microorganisms and T-cell activation/transcriptional activation on T-cells.

Overall, there are more functions controlled by DE than by AS. Many overlap and occasionally a function that was assured by DE is in a latter stage regulated also by AS. Although some immune related functions such as defense against microorganisms or T-cell activation are cooperatively achieved by AS and DEG (especially in latter stages of DC response), most of these functions are performed by genes that are differentially expressed. Alternatively spliced genes mainly target more general cellular functions. Due to statistical limitations the significance of single gene expression values for genes that have not been verified by RT-PCR or analyzed as part of a pathway should be further verified independently.

## Discussion

Alternative splicing is a ubiquitous and central mechanism for the regulation of gene expression and the generation of protein diversity. The abundance of genes in the immune system that are alternatively spliced, and the connections between splicing and disease, suggest a role for alternative splicing in the regulation and fine-tuning the function of the immune system [16]. However, there has been little effort to develop a coherent picture of how alternative splicing might be used as a general mechanism to regulate the function of the immune system.

Dendritic cell maturation following exposure to a pathogen leads to an extensive gene expression reprogramming. So far, most of the profiling studies on DCs have focused on genome-wide expression changes [26], [42], [43], [44], [45], [46], [47], [48], [49], [50], [51], [52], [53]. Our study is to our knowledge, the first that analyses collectively the expression and alternative splicing profiles as contributors to the regulation of the immune response in dendritic cells. We used GeneChip® Human Exon Arrays from Affymetrix (Affymetrix, Inc., Santa Clara, CA), that allow a genome-wide profiling, to map and describe differences in expression and alternative splicing events during an immune response in DCs challenged with *E.coli*. Our results indicate that differential expression and alternative splicing are widespread in DCs, not restricted to immune responsive genes, and are dynamically regulated by the microbial challenge over time. Such large-scale changes in expression and splicing patterns are in agreement with the unique ability of DCs to change their cellular phenotype during maturation, in response to stimuli. We found a large number of genes that underwent both AS and DE. In T-cell activation, Ip *et al.* [24] found only relatively few genes affected by both mechanisms, and that different GO terms were enriched among genes that were DE and AS. These findings can possibly be explained by a remarkable difference between these two types of immune cells: T-cell activation is a reversible process, whereas DC activation is permanent and terminal. Although our analysis of enrichment of canonical pathways and gene sets showed that overall, there were more biological processes controlled by DE than by AS, many overlapped and occasionally a function that was assured by DE is in a latter stage regulated also by AS. Many of the functional groups commonly regulated by DE and AS related to processes characteristic to DC maturation, such as cell maturation/differentiation, T-cell activation and apoptosis. We also observed that while some immune related functions were achieved also by AS most of these functions were performed by genes that are differentially expressed. Alternatively spliced genes mainly relate to more general cellular mechanisms, such as p53 and TGF-beta signaling or DNA replication.

At the expression level, we found pervasive downregulation during DC maturation, with 68% of the genes downregulated at T1 and T2. Olex *et al.* [53] found that overall more processes in DC maturation are downregulated rather than upregulated, and suggested that this indicated that DCs are preparing for cell death. In our functional analysis, transcriptional repression was assured cooperatively by AS and differential expression throughout DC maturation in response to *E.coli* challenge, emphasizing the importance and tight regulation of gene expression downregulation during DC maturation program. At T3, after 18 h of *E.coli* challenge, DCs have matured, are in migration to the lymph nodes, and preparing for entering apoptosis [26], [44]. This is compatible with the lesser extent of DEG and ASE found at this time point and also the change in the trends of expression, with fewer genes being downregulated (49% *vs* 68% at T1 and T2).

Given the lack of precedence for such a large-scale study in signal-responsive regulation of splicing in DC, we sought to validate our results in samples other than those hybridized in the arrays, derived from a pool of donors. Remarkably 60% of the results predicted by the microarray data to have an altered splicing pattern upon *E. coli* challenge were confirmed. This rate of validation compares favorably with other studies using the same array platform. These results substantiate the utility of microarray approach for predicting novel AS events associated to DC maturation and further confirm the physiological incidence of regulated AS in the immune system. Importantly our study confirmed 7 novel examples of activation induced ASE in DCs (ABTB2, CDKN3, COL2A1, CNOT6L, IRF4, SIDT2 and STAB1) with potential interest for the maturation process of these cells. We found AS between non-stimulated and stimulated DCs in Cyclin-dependent kinase inhibitor 3 (CDKN3). The isoform arising only in non-stimulated cells has been formerly described. However we also found two novel isoforms in both stimulated and immature cells that may play a physiological role in these cells. CDKN3 belongs to the protein phosphatases family and is involved in regulating the cell cycle [54], [55]. It has a dual function in cell cycling: acting upstream of the transcription factor E2F1 and preventing the generation of proteins required for G1/S transition [56],[57] thereby inhibiting G1/S transition [58] or by abolishing the induction of p21 thus facilitating cell cycle progression [59]. It will be interesting to assess how the differential splicing of CDKN3 in DCs upon stimulus may regulate cell cycle progression, or other functions in the immunobiology of DCs. Another gene that may be participating in this particular feature is CNOT6L. CNOT6L was identified along with CNOT6 as a key regulator of insulin-like growth factor–binding protein 5 (IGFBP5), which mediates cell cycle arrest and senescence via a p53-dependent pathway [60]. It has been reported that in eukaryotic cells, the poly(A) tail of most mRNAs in the cytoplasm gradually get shorter, and mRNAs with shorter poly(A) tail are translated less and degraded sooner [61]. The Ccr4-Not complex is one of the major deadenylase factors present in eukaryotic cells, and CNOT6L is one of the subunits of this complex [62]. Thus, additionally to a role in the cell cycle, the novel isoform of this gene we found only in stimulated DCs may also be contributing to the extensive downregulation program seen throughout DC maturation.

Proteolysis in the endo/lysosomal compartment generates peptides that bind and are displayed by class II MHC molecules reflecting the extracellular environment for effective survey by CD4+ T cells [63]. This process of antigen processing and presentation is used to display foreign and self peptides (when sampling apoptotic cells) and is therefore important for ‘self’ tolerance as well as immunity to pathogens [63]. We found AS in SID1 transmembrane family, member 2 (SIDT2), that was recently identified as a highly glycosylated lysosomal integral membrane protein [64]. It has been suggested that glycoproteins localized at the limiting membrane of lysosomes may have a role in MHC II-restricted antigen presentation [65]. This alternative splicing event might have a functional relevant role in antigen presentation. This is also the case for the multifunctional scavenger receptor, Stabilin-1 (STAB1), involved in complex physiological clearance processes [66], [67], for which we found a novel isoform differentially spliced between non-stimulated and stimulated DCs.

To systematically investigate the extent of splicing regulation, we were particularly interested in the results obtained while analyzing a comprehensive list of SFs (Table S5). It has been shown that SF gene expression signatures can be identified, that correlate with tissue-specific patterns of alternative splicing [68]. By performing exon and gene expression profiling in the same dataset, de la Grange *et al.* [69] showed that the prevalence of alternative splicing in the cerebellum and testis is likely to originate from a larger number of genes, including genes coding for SFs that are more expressed in these tissues. Similarly we established that in our cells, larger percentages of the splicing factors were expressed, when compared to the other genes, probably contributing for the significant number of ASE we found. Among the differentially expressed SFs, the majority had low logFCs. This is in agreement with the results of Olex *et al.* [53], who report that many important characteristics of DC maturation may be found in large numbers of genes that exhibit downregulation with expression changes between 2–4 fold. We verified that SFs are among those genes. This group studied the temporal dynamics of DC maturation by stimulating mouse DCs with poli(I:C), and assessed gene expression changes at different timepoints. Although their studies focused on a different species, they found 19 out of the 71 SFs we established to be DE, to follow a similar trend of expression. The two most affected protein families within differentially expressed SFs are snRNPs and spliceosome associated proteins. SnRNPs are RNA-protein complexes that combine with unmodified pre-mRNA and various other proteins (the SAPs) to form a spliceosome [70]. Sequence-based predictions have revealed that approximately one-third or more of AS events have the potential to introduce a premature termination codon (PTC) that could target the resulting spliced transcript for nonsense-mediated mRNA decay (NMD) [71]. AS-coupled NMD is likely involved in the regulation of core spliceosomal components or assembly factors. Saltzman *et al.* [72] performed a computational search for AS events in a list of spliceosomal factor genes and found that conserved, PTC-introducing AS events were significantly enriched in these genes, indicating that proteins involved in the formation of the core spliceosome are regulated by AS-coupled NMD and the results implicated these proteins in autoregulatory loops [72]. Given that a large percentage of the SFs is expressed in DCs, downregulation is not equivalent to non-expression, but to less expression in low FCs. We propose that there may be a double regulation for some of these spliceosomal genes, coupling downregulation with NMD, reflecting the tight AS regulation required in DC maturation process.

The preponderance of the DE among SFs is seen in the early and mid stage (Figure 2b). Only 4 SFs are newly differentially expressed after 18 h of challenge (though 273 are expressed), coinciding with a lower percentage of genes being alternatively spliced. Interestingly a larger percentage of the SFs is upregulated at this timepoint (50% *vs* 6–18% at T1/T2), probably in relation to a program required for a terminally differentiated dendritic cell, at this point involved in antigen presentation.

Distinct yet sometimes overlapping functional groups were observed in DEG and ASE suggesting that regulation of AS is a broad mechanism that operates in concert with regulated transcription, in order to provide an appropriate DC response to stimulus. Thirty one to 38% of the alternatively spliced genes are also differentially expressed and 21–27% of the DEG are alternatively spliced as well. At all timepoints several genes undergo more than one splicing event (ASE>AS genes), suggesting several layers of regulation for each gene. A dynamic process of interaction between transcripts, regulatory sequences and alternative splicing events creates an underlying gene expression network that is extremely important for controlling many of the changes observed during DC maturation. We have identified a SF specific signature with tight regulation in these cells. It will be interesting to assess how they coordinate groups of ASE in DCs. Also an exhaustive identification of the functions of the identified isoforms will be required, to determine the specific consequences AS in DCs.

## Supporting Information

Figure S1 **Genes without perceptible alternative splicing in dendritic cells after challenge with** ***E.coli*** **.** Analysis of PCR products on GelRed stained 1.5–2% agarose gels (in some cases the figure was cropped so that the lane with the DNA ladder was adjacent to the lanes of interest) and schematic representation of the region of the gene that was tested. The yellow asterisk marks the significant probeset and the blue boxes represent the areas where the primers were designed to anneal. The same amount of cDNA was used in all RT-PCR reactions, as shown in GAPDH amplification reaction. Details are supplied in Table S2.
(TIF)

Table S1 **Genes used for qPCR validation of the exon-arrays analysis, concerning gene expression.**
(DOC)

Table S2 **Validation of Alternative Splicing Events description.**
(XLS)

Table S3 **Differentially Expressed Genes.**
(XLS)

Table S4 **Comparison of Huang** ***et al.*** **'s data with ours.**
(XLS)

Table S5 **Splicing Factors.**
(XLS)

Table S6 **Differentially Expressed Splicing Factors.**
(XLS)

Table S7 **Alternative Splicing Events.**
(XLSX)

Table S8 **Alternatively Spliced Splicing Factors.**
(XLS)

Table S9 **Functional Analysis.**
(XLS)
