# Supplementary figures and images for: Genome-Wide Analysis of Alternative Splicing during Dendritic Cell Response to a Bacterial Challenge
Source: PLoS One. 2013 Apr 17;8(4):e61975. doi: 10.1371/journal.pone.0061975 (PMC3629138; doi:10.1371/journal.pone.0061975)

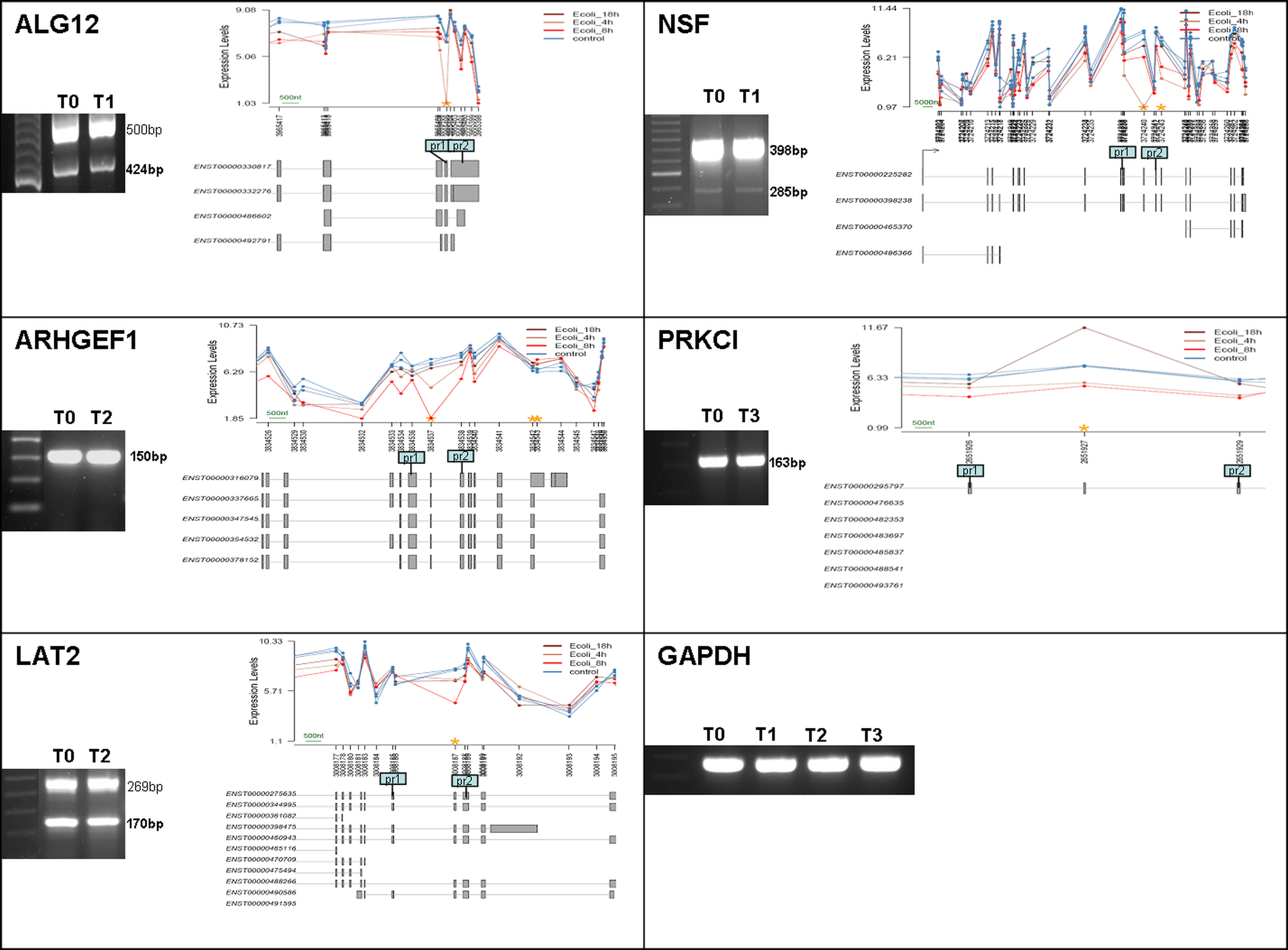

Supplement: Figure S1 — Genes without perceptible alternative splicing in dendritic cells after challenge with E.coli . Analysis of PCR products on GelRed stained 1.5–2% agarose gels (in some cases the figure was cropped so that the lane with the DNA ladder was adjacent to the lanes of interest) and schematic representation of the region of the gene that was tested. The yellow asterisk marks the significant probeset and the blue boxes represent the areas where the primers were designed to anneal. The same amount of cDNA was used in all RT-PCR reactions, as shown in GAPDH amplification reaction. Details are supplied in Table S2. (TIF) [file pone.0061975.s001.tif]
