# Supplementary material for: Genome-Wide Analysis of Alternative Splicing during Dendritic Cell Response to a Bacterial Challenge
Source: PLoS One. 2013 Apr 17;8(4):e61975. doi: 10.1371/journal.pone.0061975 (PMC3629138; doi:10.1371/journal.pone.0061975)
Supplement: Table S1 — Genes used for qPCR validation of the exon-arrays analysis, concerning gene expression. (DOC) [file pone.0061975.s002.doc]

**Table S1** - Genes used for qPCR validation of the exon-arrays analysis, concerning gene expression.

| **Gene** | **logFC in DCs - Arrays** | **logFC in DCs - qPCR** | **Validation timepoint** | **Forward Primer (5’--3’)** | **Reverse Primer (5’--3’)** | **SF** | **Reported in Huang et al. (2001)** |
| --- | --- | --- | --- | --- | --- | --- | --- |
| **CCL5** | 5.26 | 5.58 | T2 | GCGCTCCTGCATCTGCCTCC | CCTTGATGTGGGCACGGGGC |  | YES |
| **CSDA** | 2.28 | 0.91 | T3 | TGCCATCAAGAAGAATAACC | ATCTGCAGCGTAACGACTC | YES | YES |
| **CXCL1** | 6.16 | 8.61 | T1 | CCTGCTCCTGGTAGCCGCTG | TTCGGTTTGGGCGCAGTGGG |  | YES |
| **CXCL2** | 3.10 | 6.07 | T1 | CCCCTGGCCACTGAACTGCG | GGGCGATGCGGGGTTGAGAC |  | YES |
| **CXCL3** | 3.15 | 6.93 | T2 | TCTCCCGCTTCTCGCACAG | GCGGGGTTGAGACAAGCTTT |  | YES |
| **DHX35** | -2.37 | -2.68 | T1 | CAGGACTGCCTTCCTTTGAG | TAGCTGATGCCTGGGAGACT | YES |  |
| **EIF2AK2** | 2.71 | 3.32 | T1 | ACGCTTTGGGGCTAATTCT | TTCTCTGGGCTTTTCTTCCA |  | YES |
| **EXOSC2** | -2.88 | -1.53 | T2 | CTGCATCATCTCGCTGGTAA | CATATGGGGACCACACATCA | YES |  |
| **IFIT3** | 7.10 | 4.55 | T1 | TGAGCCGTGCCGTGCGTGAG | GCCGCTTGATCTTGGACGA |  | YES |
| **IL1B** | 7.54 | 7.20 | T2 | CTCGCCAGTGAAATGATGGCT | GTCGGAGATTCGTAGCTGGAT |  | YES |
| **IL8** | 5.88 | 6.76 | T1 | ACTGAGAGTGATTGAGAGTGGAC | AACCCTCTGCACCCAGTTTTC |  | YES |
| **IMP3** | -4.15 | -2.06 | T1 | TGAGCCGTGCCGTGCGTGAG | GCCGCTTGATCTTGGACGA | YES |  |
| **INTS3** | -1.89 | -0.92 | T1 | CAGGCCTTTACCCCACTGTA | TATCGGGATCTCTGGATTGC | YES |  |
| **MOV10** | 1.66 | 2.19 | T3 | TTCTACATTGCCCGCTTCT | CCAGCGCCATACTTAACTCC | YES |  |
| **NHP2L1** | -4.26 | -2.47 | T2 | ACAAGAATGTGCCCTACGTG | GGTGACAGAACAGGCGATG | YES |  |
| **NFKB1** | 3.45 | 4.94 | T1 | CCTGGATGACTCTTGGGAAA | TCAGCCAGCTGTTTCATGTC |  | YES |
| **PARP1** | -3.04 | -1.60 | T2 | TCGAGTCGAGTACGCCAAGA | GGCCATCCGGAGCGAGTC | YES |  |
| **PIM1** | 3.48 | 2.57 | T2 | GTCCAAAATCAACTCGCTTGC | CCACCTGGTACTGCGACTC |  | YES |
| **PPIL3** | -3.00 | -1.58 | T2 | TAATAATGGCCCGAACAC | CCGGGTAAACCACAATAAGT | YES |  |
| **PRPF19** | -1.66 | -0.73 | T2 | GGAAGACTGTGCCTGAGGAG | CCCACCAGTGAGGATCTTGT | YES |  |
| **PTGS2** | 8.04 | 9.57 | T1 | GTGCAACACTTGAGTGGCTAT | AGCAATTTGCCTGGTGAATGAT |  | YES |
| **RBM26** | -2.41 | -1.27 | T1 | CTCCACTTCCTCCTTTGCAG | CTTGGGGCTTCAGGATTGTA | YES |  |
| **S100A9** | 4.02 | 1.49 | T3 | AGCTGGAACGCAACATAGAG | TTGGCCACTGTGGTCTTAG | YES | YES |
| **SMNDC1** | 2.40 | 0.99 | T2 | GACGCTTGCAAGTTCAGACA | CCTTTGCCTTCCTTCCTTCT | YES |  |
| **SRPK2** | -2.58 | -1.37 | T2 | AGGACCCTGCGGACTACTG | GGGAGGCCTTGATAGTTGG | YES |  |
| **STAT2** | 1.90 | 2.39 | T2 | GAGGCCTCAACTCAGACCAG | GCGTCCATCATTCCAGAGAT |  |  |
| **TNFa** | 5.57 | 3.89 | T2 | CCGAGTGACAAGCCTGTAGC | GAGGACCTGGGAGTAGATGAG |  | YES |
| **WTAP** | 1.76 | 2.12 | T3 | TTGGAGGGCAAGTACAC | AAGTTGATCGCTGGGTCTAC | YES |  |
| **GAPDH** |  |  |  | GAGTCAACGGATTTGGTCGT | TTGATTTTGGAGGGATCTCG |  |  |

logFC- log_2_Fold change, SF – Splicing factor
